# Bilateral Destructive Hip Disease from Untreated Juvenile Idiopathic Arthritis

**DOI:** 10.1155/2019/4593129

**Published:** 2019-03-06

**Authors:** Arash Calafi, Alton W. Skaggs, Trevor J. Shelton, Brian M. Haus

**Affiliations:** ^1^University of Washington Department of Orthopaedics and Sports Medicine, 1959 N.E. Pacific St., Box 356500 Seattle, WA 98195, USA; ^2^School of Medicine at University of California at Davis, Sacramento, CA 95817, USA; ^3^Department of Orthopaedic Surgery, University of California at Davis, 4860 Y Street, Suite 3800, Sacramento, CA 95829, USA; ^4^Shriners Hospital for Children, Northern California, 2425 Stockton Blvd., Sacramento, CA 95817, USA

## Abstract

We report a novel case of a pediatric patient with bilateral hip destruction from untreated Juvenile idiopathic arthritis (JIA). She was presented at the age of 9 with hip pain associated with bilateral acetabular dysplasia and a dislocated left femoral head. Only 1.5 years later, the patient developed complete destruction of the left femoral head and dislocated right femoral head. The authors have not identified literature describing a similar case report of bilateral femoral head destruction resulting from Persistent Oligoarticular JIA. Pediatric patients presenting with rapidly evolving destructive process should be evaluated for rheumatologic, infectious, and spinal etiologies.

## 1. Introduction

Juvenile idiopathic arthritis (JIA) is an umbrella term used to describe arthritis of unknown origin in greater than one joint, lasting greater than 6 months in patients with disease onset prior to age of 16 [1]. JIA is a diagnosis of exclusion, considered only after more common etiologies including septic joint, trauma, malignancy, or other immunologic disorders are ruled out. Presently, JIA consists of six categories with distinct clinical and laboratory features (Table 1) [2].

While specific pathophysiologic processes of JIA categories may differ, T-cell-mediated destruction of joint spaces leading to a significant loss of function, pain, stiffness, fatigue, joint effusion, reduced range of motion, erythema, and growth alterations is the prevailing cause of morbidity in these patients [3, 4]. Impacted joints of serious consequence are the knee, hip, and temporomandibular joint and may require surgical intervention [5, 6]. Hip involvement in those with JIA is reported between 20%-40%, and early identification of hip involvement in these patients may lead to more aggressive early treatment before hip joint destruction occurs [7]. However, when it does occur at a young age, it becomes challenging to treat and manage. Here, we present a case of a 10-year-old Mexican female who developed destructive bilateral hip disease in the setting of untreated Persistent Oligoarticular JIA.

### 1.1. Case Presentation

The patient was initially presented to our clinic at the age of 9 with a chief complaint of limping. Her developmental history was notable for developmental delay without underlying genetic or autoimmune conditions and low infantile weight for which she had a gastric tube. Family history was negative for any neuromuscular disease, hip dysplasia, or autoimmune disease. She had recently moved from Mexico with her family to the United States.

**Figure 1:**
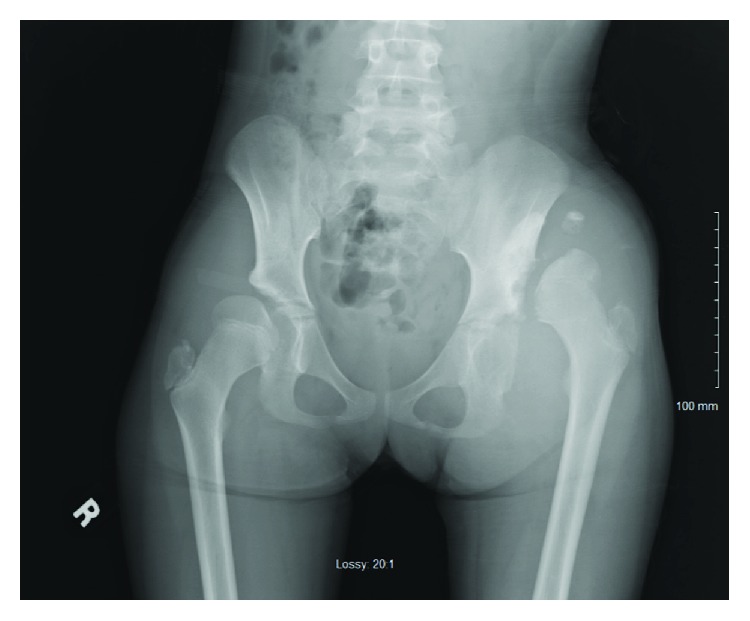
Plain AP radiograph performed in April 2014 shows dislocation of the left hip and pseudoacetabulum.

**Figure 2:**
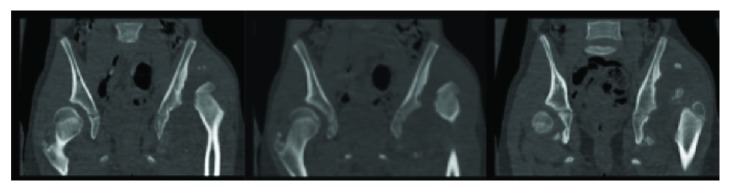
Coronal cuts of CT scan shows bilateral acetabular dysplasia with dislocated left femoral head localized in the anterosuperior aspect of the acetabulum with development of pseudoacetabulum.

Her initial exam revealed a Trendelenburg gait and sign, left hip pain-free range-of-motion with 100° internal rotation, 50° external rotation, 135° flexion, 20 extension, and 70° abduction. Her right hip revealed pain-free range-of-motion with 90° internal rotation, 50° external rotation, 135° flexion, and 60° abduction. The comparison of both hips revealed a positive Galeazzi sign with a leg-length difference of 2 cm and normal bilateral reflexes. Imaging demonstrated bilateral hip dysplasia with left hip dislocation with pseudoacetabuli (Figures 1 and 2). At this time, the working diagnosis was untreated bilateral hip dysplasia, and we discussed surgical options including fusion, pelvic and femoral osteotomies, and total hip arthroplasty (THA). Given the chronicity of her dislocation and severity of her dysplasia, it was determined that any surgical treatment on the unilateral left hip dislocation was unlikely to provide a significant benefit and may make her worse. We recommended proceeding with a possible THA versus fusion in her teenage years when pain warranted treatment. For the right hip, we recommended a future periacetabular osteotomy (PAO) once her triradiate cartilage closed and her hip became symptomatic, possibly in a few years.

**Figure 3:**
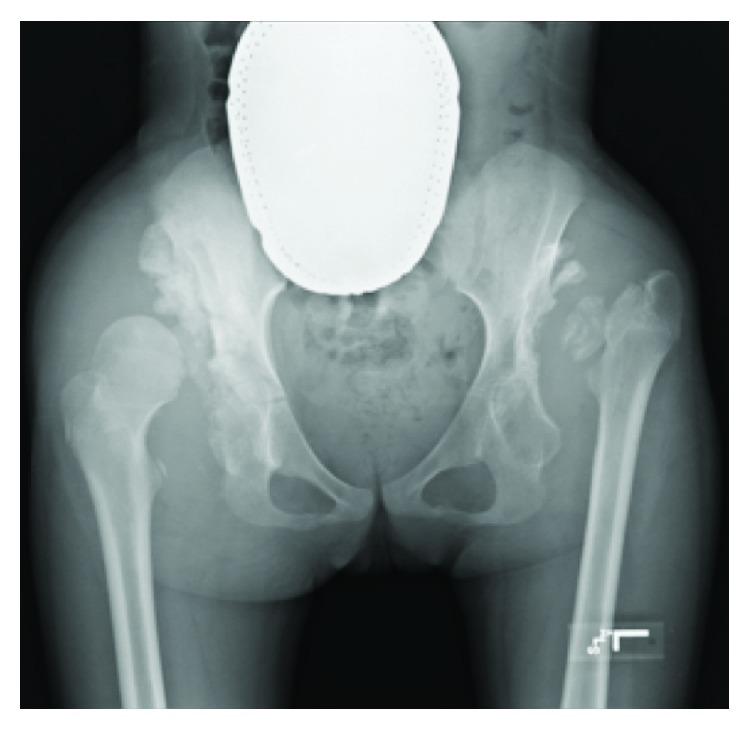
Plain AP radiograph performed on admission shows bony destruction of the bilateral acetabula and rapid progression of the left proximal femur with early cortical erosion of the right femoral head.

**Figure 4:**
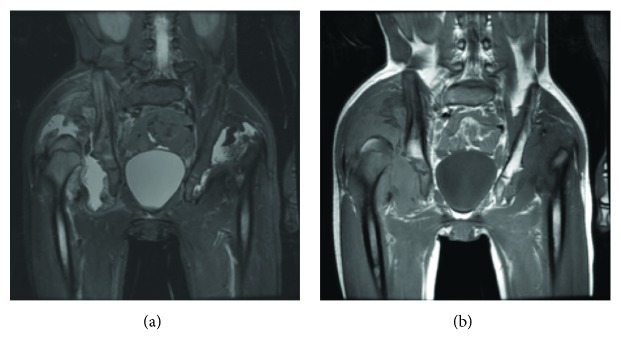
MRI pelvis with coronal T1 (a) and coronal STIR (b) of pelvis performed on admission shows bilateral dislocated hips with right greater than left joint effusions, synovial thickening, and rice body formation.

She was unfortunately lost to follow-up and did not receive additional treatment during this time but represented to our clinic 1.5 years later with progressive decrease in ambulation requiring a wheelchair and walker and bilateral left greater than right hip pain. On exam, both hips had painful range-of-motion with the development of a 5° hip flexion contracture on the left and increased flexion, abduction, and internal rotation on the right. Her limp was significantly worse, but her leg-length discrepancy decreased to 0.5 cm. Imaging demonstrated erosive changes of the bilateral femoral heads with infiltrative destructive patterns to the bilateral acetabula, hip effusions, and synovial thickening (Figures 3 and 4). In addition, she developed a new dislocation of the right hip. She was directly admitted from clinic for further work-up. On the day of admission, she had bilateral hip aspirations which showed unremarkable inflammatory fluid changes (left hip: 20 mL of cloudy, turbid, straw-colored fluid, cell count 908, polymorphonuclear cells 2%; right hip: 80 mL of bloody, cloudy fluid, cell count 359, polymorphonuclear cells 49%). Crystal exam, gram stain, and cultures were negative. Synovial fluid cytology demonstrated numerous benign reactive synovial cells without identification of malignant cells.

Given the severity of the destruction, a possible spinal etiology including tumor syrinx resulting in Charcot joint was suspected. Magnetic resonance imaging of the brain and cervical/thoracic/lumbar spine was within normal limits. Other etiologies including bacterial, fungal, and viral infection were considered. Coccidioidomycosis and histoplasmosis titers and Lyme disease antibodies were negative. To evaluate for tuberculosis, a chest X-ray was obtained within normal limit while CT demonstrated a 4 mm nodule in the right middle lobe that was noncalcifying and believed to be postinfectious. AqQuantiFERON-TB gold test was negative. A complete blood count, basic metabolic panel, CRP (0.3 mg/dL), and ESR (7 mm/hr) were within normal limits with the exception of elevated platelets (550). Compliment fixation serum and immunodiffusion serum tests were negative. Autoimmune etiologies were also considered. Extensive serologic testing was performed including dilute Russel's viper venom time, lupus anticoagulant, anti-CCP, anticentromere, antimitochondrial, anti-double-stranded DNA, antinuclear antibody, anti-SSA, SSB antibody panel, rheumatoid factor (RF), and HLA-B27 which were all negative.

**Figure 5:**
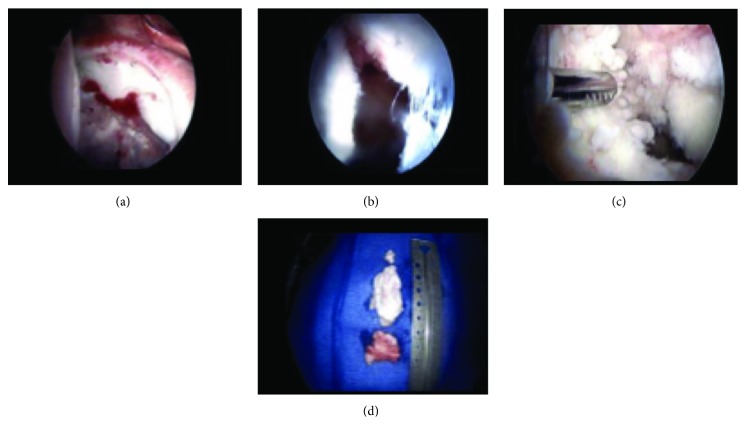
Left hip arthroscopic imaging shows (a) intact labrum with destruction of the acetabulum, (b) destroyed joint capsule, (c) cartilaginous destruction of the femoral head and acetabulum, and (d) loose bodies removed from the left hip.

**Figure 6:**
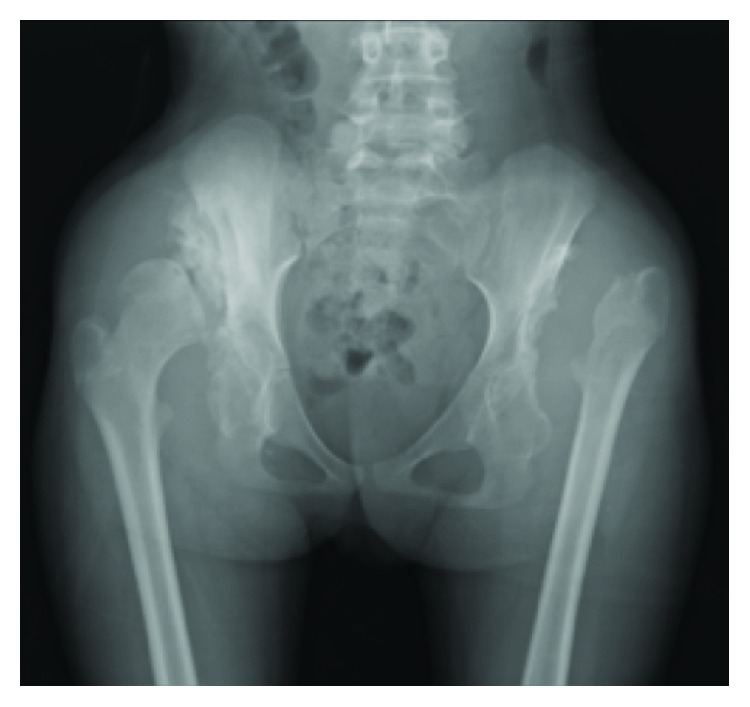
Plain AP radiograph performed 6 weeks after surgery shows interval resection of the femoral head that was fragmented. There is also resection of bony fragmentation adjacent to the acetabulum.

Because of the unusual and severe presentation of the disease and the lack of a diagnosis, on hospital day 10, she was taken to the operating room for bilateral hip arthrocentesis and bilateral hip arthroscopy for biopsy of the bone and synovium. A synovial biopsy was obtained to examine the tissue visually and histologically to confirm the diagnosis of an inflammatory arthritis. Intraoperative findings demonstrated right capsular destruction with unusual appearing and inflamed and reddened synovium with erosive destruction of the articular surface of the femoral head and acetabulum. On the left, there was a free-floating femoral head with multiple moderate-sized osteochondral loose bodies grossly inflamed synovium, intact labrum, no clearly discernable joint capsule, and near-complete absence of cartilage within the acetabulum and overlying the femoral head (Figure 5). Following the diagnostic component of the arthroscopy, she underwent a left femoral head arthroscopic resection, bilateral synovial and bone biopsies, and bilateral Kenalog intra-articular injections for pain relief. Biopsy results demonstrated synovial hyperplasia with chronic inflammatory changes. Postoperative X-rays showed left femoral head resection (Figure 6).

Given the results of the arthroscopic findings and synovial biopsy, we subsequently referred the patient to a board-certified pediatric rheumatologist for further evaluation. Additionally, she was found to have moderate arthropathy of her right calcaneocuboid and bilateral 1^st^ metatarsophalangeal joints. Given these clinical findings, she was given the diagnosis of Persistent Oligoarticular JIA characterized by negative RF, CCP, and HLA-B27 and was subsequently started on methotrexate and folic acid.

**Figure 7:**
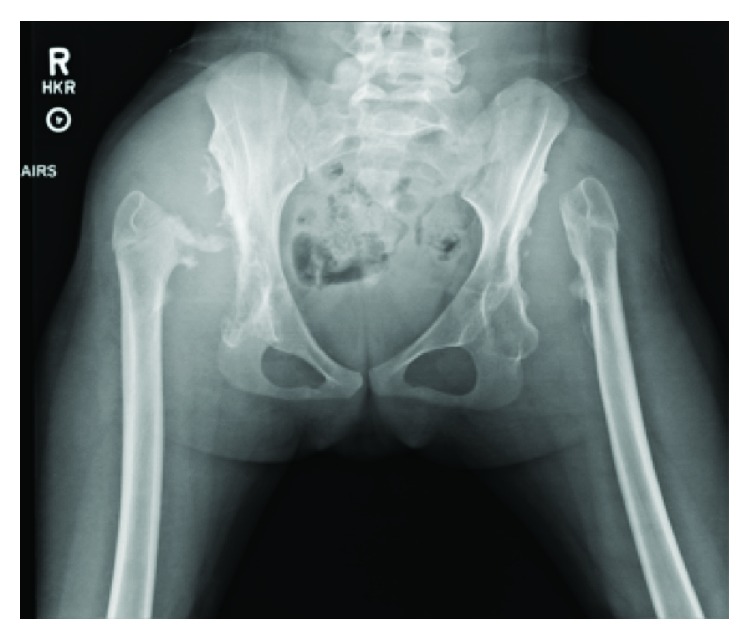
Plain AP radiograph performed one year after arthroscopic removal of femoral heads shows interval erosion of the right femoral head.

At 3-year postoperatively, she has continued erosion of the right femoral head (Figure 7). A genetics consult was obtained, and comprehensive screening was negative. She remains nonweight bearing with the plan for bilateral THAs once she becomes a teenager. However, this could result in multiple challenges if the patient becomes nonambulatory, has limited acetabular bone stock, if the hip dysplasia requires femoral shortening, or if custom implants are needed due to the small size and deformity.

## 2. Discussion

Oligoarticular JIA (≤4 joints involved) is the most common subgroup of JIA and constitutes approximately 50% of JIA cases. It is subdivided into persistent oligoarthritis (no additional joint involvement after the first six months) and extended oligoarthritis (involvement of additional joints after the first six months) [2]. Typically, oligoarticular JIA affects females more than males, rarely presents after 10 years of age and generally affects large joints (most commonly knees and ankles) [8]. Initial symptoms in the hip are very rare in oligoarticular JIA and should raise concern for other etiology including Legg-Calve-Perthes, infection, spondyloarthropathy, neoplasm, or slipped capital femoral epiphysis. Although the hips are rarely involved, when they are, it is associated with a poor prognosis prior to starting treatment [9]. The diagnosis of oligoarticular JIA is made based on the presence of arthritis in ≤4 joints during the first six months of the disease after other causes have been excluded [2].

Treatment of oligoarticular JIA requires a multidisciplinary approach involving rheumatologist, physiotherapy, psychosocial support, pharmacologic therapy, and if warranted, surgical intervention. However, nearly 50% of the patient's symptoms will persist into adulthood [10]. Therefore, pharmacologic treatment prioritizes early and aggressive preservation of joint tissue. As such, disease-modifying antirheumatic drugs are the mainstay of JIA treatment. In addition, agents to control inflammation such as nonsteroidal anti-inflammatory drugs and corticosteroids are commonly prescribed. Biologics, including anti-TNF agents, may also be useful in patients with refractory JIA [1, 11]. Close follow-up is therefore essential, as combined immunosuppressive agents increase the risk of serious opportunistic infections.

Approximately 22% of patients with JIA will require major surgical intervention during their lifetime [12]. From 1991 to 2005, the average age of joint replacement performed in patients with JIA increased from 30.9 to 36.7 while the overall rate of arthroscopies dropped nearly 50% [13]. This trend is likely from more aggressive and early use of immunomodulating agents, helping to slow the disease course. While the knee, elbow, and ankle are more commonly affected in patients with JIA, hip destruction is associated with greater functional deficits and morbidity [14]. Hip involvement in those with JIA is 20%-40%, but it is rarely the initially affected joint. Rather, hip symptoms typically manifests within 7 years after the initial diagnosis [7]. Typically, with the aid of immunomodulating agents, patients do not develop hip destruction necessitating joint replacement until the third or fourth decade [15]. Known predictors of early hip destruction include increased joint involvement, systemic symptoms associated with JIA, elevated immunoglobulins, increased platelet count, and hip involvement early in the disease course [7].

Patients with hip destruction and severe impairment may require a THA if physiotherapy and pharmacologic treatments fail. However, optimal timing for THA is controversial. It has been shown that implant survival is significantly better in young patients over the age of 25 at the time of THA [16]. THA in younger patients is technically challenging and complicated due to several factors. The femoral medullary canal can be excessively narrow and fragile, increasing the risk for fracture and prosthetic failure. There may also be deformities such as femoral and acetabular anteversion as well as the presence of flexion contractures. Moreover, bone stock can be depleted due to immunologic destruction and prolonged use of corticosteroid [5, 6, 17]. The use of immunosuppressants also increases the risk of postoperative infection and poor wound closure [14]. Finally, when considering THA in younger patients, growth status must also be considered. Premature physeal closure and growth retardation are common in JIA due to pain-related disuse of the affected limb, cachexia secondary to chronic inflammation (increased TNF-a and IL-1), osteopenia from chronic corticosteroid use, and malnutrition secondary to increased metabolic demand and pharmacologic side effects [4, 18]. Despite these risks, THA is considered a reasonable treatment modality in patients with refractory JIA and is associated with significant improvement in joint pain and mobility with a 10-year implant survival rate of 80-85% and 20-year implant survival of approximately 35% [16].

## Figures and Tables

**Table 1:** Six categories and definitions of juvenile idiopathic arthritis [2].

Categories of juvenile idiopathic arthritis	Definitions
Systemic arthritis	Arthritis in one or more joints with or preceded by fever ≥ 2 weeks' duration and accompanied by one or more of the following: (1) Evansecent (nonfixed) erythematous rash (2) Generalized lymph node enlargement (3) Hepatomegaly and/or splenomegaly (4) Serositis

Psoriatic arthritis	Arthritis and psoriasis or arthritis and ≥2 of the following: (1) Dactylitis (2) Nail and pitting or onycholysis (3) Psoriasis in a first-degree relative

Enthesitis-related arthritis	Arthritis and enthesitis or arthritis or enthesitis with ≥2 of the following: (1) Presence of or a history of sacroiliac joint tenderness and/or inflammatory lumbosacral pain (2) Presence of HLA-B27 antigen (3) Onset of arthritis in a male >6 years of age (4) Acute (symptomatic) anterior uveitis (5) History of ankylosing spondylitis, enthesitis-related arthritis, sacroiliitis with inflammatory bowel disease, Reiter's syndrome, or acute anterior uveitis in a first-degree relative

Oligoarticular	Arthritis affecting 1-4 joints during the first 6 months of disease. Two subcategories: Persistent oligoarthritis: affecting ≤4 joints throughout the disease course Extended oligoarthritis: affecting >4 joints after the first 6 months of disease

Polyarthritis	Rheumatoid factor negative: arthritis affecting ≥5 joints during the first 6 months of disease; RF test is negative Rheumatoid factor positive: arthritis affecting ≥5 joints during the first 6 months of disease; 2 or more tests for RF at least 3 months apart during the first 6 months of disease are positive

Undifferentiated arthritis	Arthritis that fulfills criteria in no category or in 2 or more of the above categories
